# Adenylate kinase 2 (AK2) promotes cell proliferation in insect development

**DOI:** 10.1186/1471-2199-13-31

**Published:** 2012-09-28

**Authors:** Ru-Ping Chen, Chun-Yan Liu, Hong-Lian Shao, Wei-Wei Zheng, Jin-Xing Wang, Xiao-Fan Zhao

**Affiliations:** 1 The Key Laboratory of Plant Cell Engineering and Germplasm Innovation, Ministry of Education; Shandong Provincial Key Laboratory of Animal Cells and Developmental Biology, School of Life Sciences, Shandong University, Shandong, Jinan, 250100, China; 2 Hubei Key Laboratory of Insect Resource Application and Sustainable Pest Control and Institute of Urban and Horticultural Pests, College of Plant Science and Technology, Huazhong Agricultural University, Hubei, Wuhan, 430070, China

**Keywords:** *Helicoverpa armigera*, Adenylate kinase 2 (AK2), Cell growth and viability, RNA interference

## Abstract

**Background:**

Adenylate kinase 2 (AK2) is a phosphotransferase that catalyzes the reversible reaction 2ADP(GDP) ↔ ATP(GTP) + AMP and influences cellular energy homeostasis. However, the role of AK2 in regulating cell proliferation remains unclear because AK2 has been reported to be involved in either cell proliferation or cell apoptosis in different cell types of various organisms.

**Results:**

This study reports AK2 promotion of cell proliferation using the lepidopteran insect *Helicoverpa armigera* and its epidermal cell line HaEpi as models. Western blot analysis indicates that AK2 constitutively expresses in various tissues during larval development. Immunocytochemistry analysis indicates that AK2 localizes in the mitochondria. The recombinant expressed AK2 in *E. coli* promotes cell growth and viability of HaEpi cell line by 3-(4, 5-Dimethylthiazol-2-yl)-2, 5-diphenyltetrazolium bromide (MTT) assay. AK2 knockdown in larvae by RNA interference causes larval growth defects, including body weight decrease and development delay. AK2 knockdown in larvae also decreases the number of circulating haemocytes. The mechanism for such effects might be the suppression of gene transcription involved in insect development caused by AK2 knockdown.

**Conclusion:**

These results show that AK2 regulates cell growth, viability, and proliferation in insect growth and development.

## Background

Various cellular functions need ATP for energy supply. Adenylate kinase (AK or ADK) is a phosphotransferase that catalyzes the reversible reaction 2ADP(GDP) ↔ ATP(GTP) + AMP in the presence of Mg^2+^ and influences cellular energy homeostasis and cellular adenine nucleotide metabolism [1,2]. The widespread and highly conserved distribution of AK has been reported in organisms from prokaryotes to eukaryotes. Prokaryotic cells [3] and eukaryotic yeasts [4] have a single-type gene of AK essential for their survival, which indicates the significance of AK in energy metabolism. Multicellular organisms have obtained diverse isoenzymes of AK through evolution. To date, eight isoenzymes of the AK family comprising AK1-AK8 have been identified in vertebrates forming a circuit in monitoring cell energy metabolism and AMP signal transduction [5]. Among these isoenzymes, AK2 is uniquely located in the mitochondrial intermembrane space distributed in the liver and kidney [6]. The unique subcellular localization suggests that AK2 plays a unique role in energy metabolism and energy transfer by regulating the ATP/ADP rate between the cytoplasma matrix and the mitochondria [7].

Several studies have been conducted on the functions of AK. AK1 knockout in mice shows compromised energy deficiency in the heart and skeletal muscles under metabolic stress [8]. The growth and development of *Caenorhabditis elegans* is suppressed after the knockdown of AK6 [9]. The knockdown of zebra fish AK2, the only type of AK in leukocytes, causes leukocyte development defects, thus indicating the importance of AK2 in leukocyte differentiation [10]. A recent study reveals that the homozygous *AK2* (−/−) larvae of *Drosophila melanogaster* ceases growth and causes death before reaching the third instar larval stage, indicating that AK2 is necessary for the growth and development of *D. melanogaster*[11].

AK2 has been reported to be involved in cell proliferation. In humans, mutations in the AK2 gene cause a profound haematopoietic defect because AK2 is required for the proliferation and differentiation of haematopoetic cells [12]. However, others report the involvement of AK2 in cell apoptosis as AK2 translocates from the mitochondrial intermembrane space into the cytosol together with cytochrome *c* during apoptosis [13]. AK2 participates in mitochondrial apoptosis by forming a complex with Fas-associating protein with death domain (FADD) and caspase 10 in the HeLa cell line, whereas AK2 knockdown attenuates apoptosis [14]. Therefore, the real function of AK2 in regulating cell growth or apoptosis remains unclear.

This study used a lepidopteran insect *H. armigera* and its epidermal cell line (HaEpi) [15] as models to investigate the roles of AK2 in cell growth and development. The results show that AK2 from *H. armigera* shares high identity with other AK2 from various animals. AK2 is constitutively expressed in various tissues during larval development and locates in the mitochondria, similar to AK2 in vertebrates. Recombinantly expressed AK2 in *Escherichia coli* increases HaEpi cell growth and viability. AK2 knockdown in larvae results in larval growth arrest, body weight decrease, developmental stage delay, and decrease of circulating haemocytes. The mechanism behind these phenomena is the suppression of gene transcription involved in insect development caused by AK2 knockdown. Results show that AK2 plays critical roles in cell proliferation and insect development.

## Results

### *Helicoverpa* AK2 shares high identity with AK2 in various animals

The cDNA of *H. armigera* AK2 is 1,013 bp in length and encodes a 242 amino acid residue protein with a molecular weight of 27 kDa. The predicted isoelectric point of *Helicoverpa* AK2 is 8.9. No signal sequence is detected. AK2 contains a core ADK domain flanked by an ATP binding domain LID. *Helicoverpa* AK2 shares higher identities with other AK2 genes from *D. melanogaster* (73%), *Anopheles gambiae* (72%), *Danio rerio* (74%), *Mus musculus* (73%), and *Homo sapiens* (72%). Phylogenetic analysis shows that AK2 from various animals cluster into two main groups: 1) AK2 from vertebrates and 2) AK2 from insects. *Helicoverpa* AK2 is clustered with insect AK2 (Additional file 1 Figure S1).

### AK2 is constitutively expressed in various tissues during larval development

**Figure 1:**
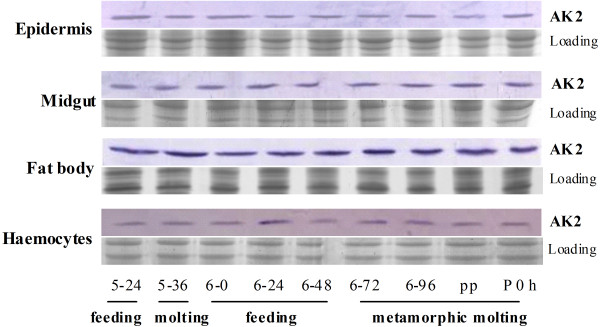
**Western blot analysis of AK2 expression profiles in tissues at different developmental stages.** 5–24 and 5–36 are the hours of 5th instar larvae after ecdysis; 6–0, 6–24, 6–48, 6–72, and 6–96 are the hours of 6th instar larvae after ecdysis; pp indicates prepupae (6th instar 120–140 h larvae); and p 0 indicates new pupae. The loading control is SDS-PAGE showing the loaded protein quantity and quality (12.5% gel).

The expression profiles of AK2 were examined in the tissues during larval development to investigate the role of AK2 in insect development. Western blot shows that AK2 expresses in various tissues, including in the epidermis, midgut, body fat, and haemocytes. The translation levels of AK2 reflect a constitutive expression in four tissues during development from the 5th instar 24 h larvae (5–24) to the 1st day pupae (p 0). These results imply that AK2 is essential during insect larval development (Figure 1).

### AK2 is co-localized with mitochondria

**Figure 2:**
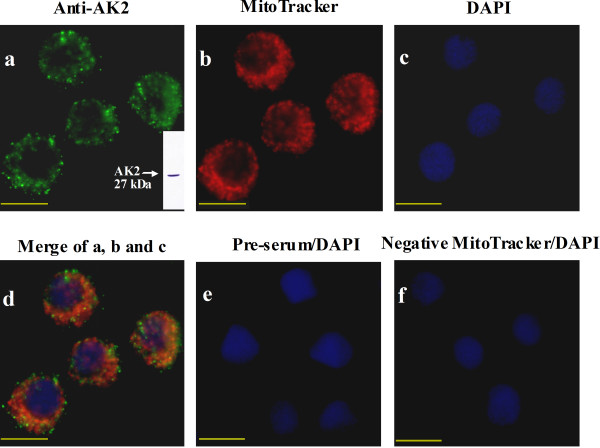
**Immunohistochemistry showing the co-localization of AK2 with mitochondria. a.** Detection of AK2 in the cells with the rabbit polyclonal antiserum against *Helicoverpa* AK2 and the goat anti-rabbit-Alexa Fluor 488, the green fluorescence indicates AK2, the column in panel a is the Western blot showing the specificity of the antibody against *Helicoverpa* AK2 using fat body from the 5th instar feeding larvae as the protein sample. **b.** Detection of mitochondria with MitoTracker, red fluorescence indicates mitochondria. **c.** DAPI-stained nucleus, blue fluorescence indicates the nucleus. **d.** Merging of images in a, b, and c, the orange color is caused by the overlap of green-colored AK2 and red-colored mitochondria. **e.** Preserum as a negative control for the antiserum against AK2. **f.** The negative control without MitoTracker. Bar is 20 μm.

Immunocytochemistry was performed in the *Helicoverpa* epidermal cell line (HaEpi) to determine the location of AK2 in the cells. Results show that AK2 locates in the cytoplasm and appears as dots (Figure 2a), implying its location in the cellular organs. Further co-localization of AK2 with mitochondria (Figure 2b) shows that AK2 is mostly overlapped with mitochondria. An orange color results from the overlap of green-colored AK2 and red-colored mitochondria (Figure 2d). Some dots of AK2 do not overlap completely with mitochondria, owing to the difference in the focus of the images from the microscope. The preserum control did not produce any green fluorescence, indicating that the antiserum against AK2 is specific to AK2 (Figure 2e). The non MitoTracker control indicates the specificity of mitochondria staining (Figure 2f). This result suggests that AK2 locates in the mitochondria.

### Recombinantly expressed AK2 in *E. coli* promotes cell growth and viability

**Figure 3:**
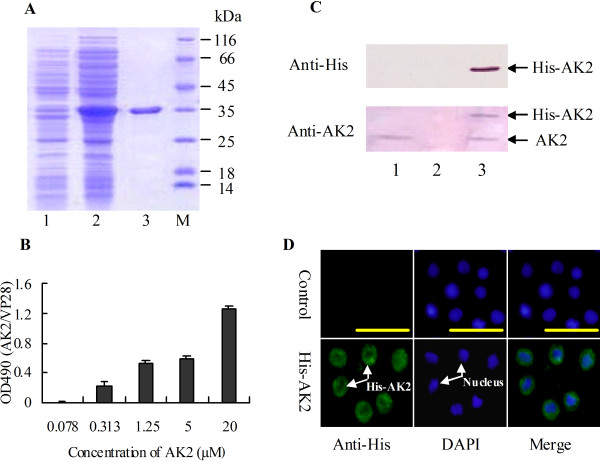
**The function of AK2 in promoting cell growth and viability. A.** SDS-PAGE (12.5% gel) showing the recombinant expression and purification of His-AK2 in *E. coli*. Lane 1, total proteins of *E*. *coli* without induction; lane 2, total proteins after induction by IPTG; lane 3, purified His-AK2; M, protein marker. **B.** MTT assay to examine the role of AK2 in cell growth and viability. *Halicoverpa* epidermal cells (HaEpi) were incubated in Grace’s medium containing different concentrations of His-AK2 (0.078, 0.313, 1.25, 5, and 20 μM) for 72 h. **C.** Western blot showing that His-AK2 entered the HaEpi cells. Lane 1, cells incubated without His-AK2; lane 2, PBS buffer from the supernatant after washing the His-AK2-incubated cells; lane 3, cells incubated with 5 μM His-AK2 for 48 h after washed by PBS. **D.** Immunocytochemistry to show the entered His-AK2 in the cells 48 h post incubation. The control is the normal cells without treatment.

AK2 was expressed as a soluble His-tagged protein in *E. coli* and purified to homogeny to analyze the function of AK2 in cell growth. The molecular mass of the purified His-AK2 is about 33 kDa, comprising AK2 (27 kDa) and His-tag (6 kDa) (Figure 3A).

The purified His-AK2 was added into the Grace’s medium to culture epidermal cells (HaEpi) to assay the function of AK2. The results of MTT assay show that His-AK2 promotes cell growth and viability at concentrations from 0.313 μM to 20 μM in 72 h, compared with the His-VP28 negative control, an envelope protein of the white spot syndrome virus expressed in *E. coli* fused with His-tag (Figure 3B).

Western blot analysis shows that His-AK2 entered the HaEpi cells to promote cell growth and viability, because His-AK2 was only detected from cells incubated with His-AK2. It was present in neither the PBS buffer used in washing the His-AK2-incubated cells nor in the non-His-AK2-incubated cells (Figure 3C). To confirm the entrance of the recombinant expressed His-AK2 in the cells, immunocytochemistry was done and His-AK2 was detected in the cells by the antibody against His-tag (Figure 3D).

### *AK2* knockdown represses larval growth and development

**Figure 4:**
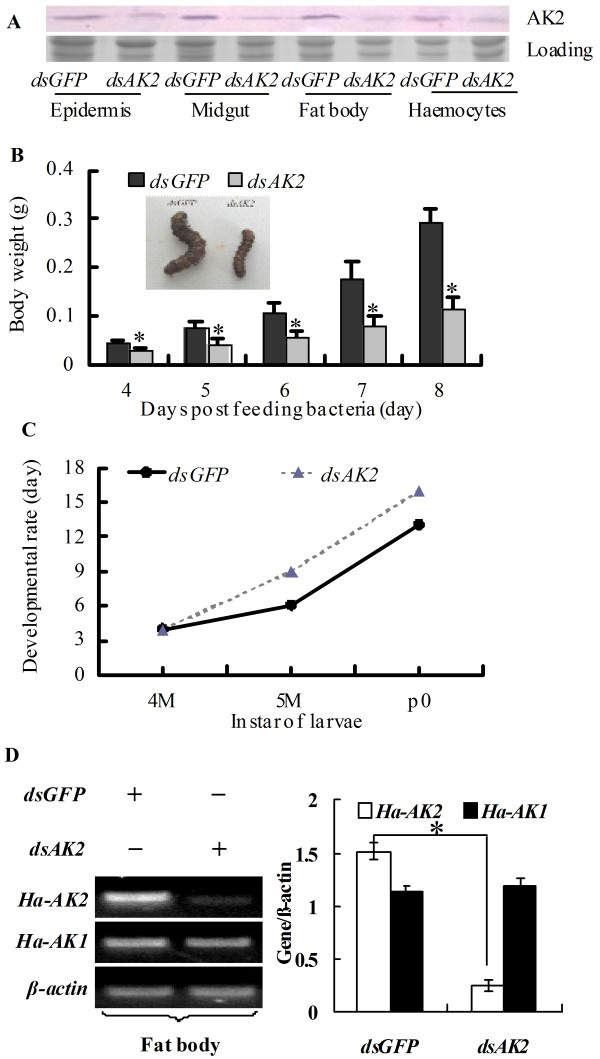
**Knockdown of *AK2 *in larvae by feeding dsRNA to newly hitched larvae suppressed larval growth and development. A.** Western blot examining the efficacy of AK2 knockdown in the larvae fed with *dsAK2*; *dsGFP* was the nonspecific dsRNA control. **B.** Statistical analysis of the lower body weight that resulted from *AK2* knockdown. **C.** Statistical analysis of the delayed developmental rate that resulted from *AK2* knockdown, 4 M, 4th instar molting toward 5th instar; 5 M, 5th instar larvae molting toward 6th instar; p0, 0 day pupae. **D.** The off target control for the AK2 RNAi, Ha-AK1 and Ha-AK2 are the *Helicoverpa* AK1 and AK2, RT-PCR (left panel), the statistical analysis (right panel). The asterisks in B and D indicate significant differences from the *dsGFP* control (p < 0.05, n = 3 × 30) by student *t* test.

*AK2* was knocked down by RNAi via continuously feeding the newly hitched larvae with *E. coli* that expresses dsRNA of *AK2* until the larvae stopped eating (6th instar 72 h) to investigate the roles of AK2 *in vivo* in insect development. Western blot results show that AK2 was knocked down systemically in various tissues, including epidermis, midgut, body fat, and haemocytes by feeding ds*AK2-*expressing *E. coli* (Figure 4A).

Due to AK2 knockdown, the larvae that fed on *dsAK2*-expressing *E. coli* exhibited growth defects and development delay, by comparison with the control that fed on *dsGFP*-expressing *E. coli*. The larvae grew slowly after knockdown of *AK2* and the body weight was lower than those of the controls (Figure 4B). In addition, the pupation time in the larvae that fed on *dsAK2* delayed about 3 days compared the control (Figure 4C). These results suggest that AK2 is necessary for larval growth and development.

To confirm that the *AK2* knockdown did not induce off-target effects on other genes, the *Helicoverpa AK1* (50% similarity to *Helicoverpa AK2*) was examined by semi-quantity RT-PCR. Results showed that *AK2* knockdown did not affect the mRNA level of *AK1* (Figure 4D), indicating that no markedly off-target effects was induced by *AK2* RNAi.

### *AK2* knockdown suppresses haemocyte proliferation

**Figure 5:**
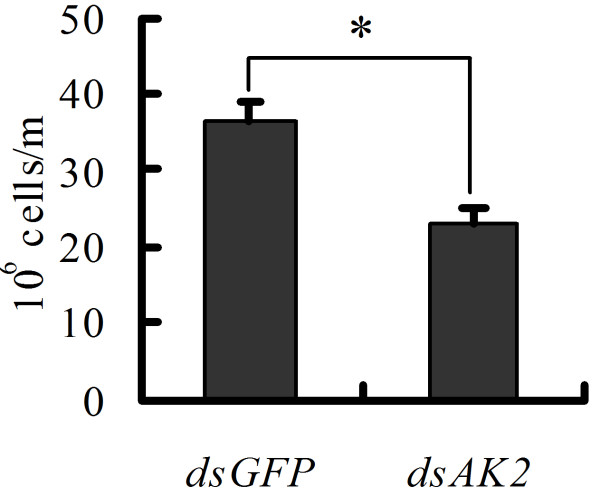
**AK2 is necessary for the proliferation of circulating haemocytes.** The cell number of haemocytes from 3 sixth instar-72 h larvae after dsRNA feeding was counted in three repetitions by blood cell count plate. The asterisk indicates significant difference from the *dsGFP* control (p < 0.05, n = 3 × 3) by student *t* test.

Circulating haemocytes were calculated after *AK2* knockdown by feeding *dsAK2*-expressing *E. coli* to the newly hitched larvae to examine the role of AK2 in cell proliferation. As expected, AK2 knockdown caused the cell number of circulating haemocytes in the 6th instar 72 h larvae decrease, compared with the *dsGFP*-expressing *E. coli* fed control (Figure 5). This finding indicates that AK2 is necessary for the proliferation of circulating haemocytes.

### *AK2* knockdown decreased the gene transcripts

**Figure 6:**
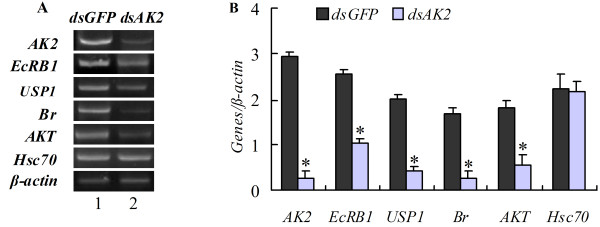
**Analysis of gene transcripts in fat body of the fifth instar molting larvae after *AK2 *knockdown by feeding dsRNA to newly hitched larvae.A.** Semi-quantitative RT-PCR, Lane 1, *dsGFP-*feeding larvae; lane 2, *dsAK2*-feeding larvae. **B.** Statistical analysis of results in A. The asterisk indicates significant difference from the control (p < 0.05, n = 3) by student *t* test.

The transcripts of some genes involved in development were examined to demonstrate the molecular mechanism of growth retardation and delayed development of larvae after *AK2* knockdown by feeding *dsAK2*-expressing *E. coli*. The results of semi-quantitative RT-PCR showed that knockdown of *AK2* caused suppression of the molting hormone nuclear receptor *EcRB1*, transcription factors *USP1* and *Br*, as well as protein kinase *AKT* genes (Figure 6). These results suggest that *AK2* knockdown decreased the gene transcripts.

## Discussion

The subcelluar localization of AK2 in the mitochondria indicates that it plays a unique role in regulating cell energy homeostasis to balance ATP/ADP between cytosol and mitochondria [6]. *H. armigera* AK2 localizes in the mitochondria, too. *H. armigera* AK2 shows high identity to other AK2 from various animals, including mammals, indicating that its function in regulating the growth and development of organisms is conserved. The current work demonstrates that AK2 promotes cell growth and proliferation in larval growth and development.

AK2 is necessary for the survival of *Drosophila*, because the lack of AK2 gene leads to growth defects and death [11]. The constitutive expression pattern of *Helicoverpa* AK2 implies that AK2 is essential for the development and growth of insect. *AK2* knockdown in *H. armigera* results in growth defects, including growth retardation, body weight decrease, and development delay. However, the larvae ultimately experienced pupae and advanced to adult. This might because that RNAi cannot deplete AK2 gene completely as the mutation in *Drosophila*. Mammalian AK2 is increased expression during adipocyte and B cell differentiation [16]. However, in *Drosophila*, the mRNA and protein of AK2 are detected in the embryo, larva, and adult [17]. *Helicoverpa* AK2 is expressed in the examined tissues consistently during development. The reason of the different expression profiles in mammals and insects might because the difference between the organisms.

AK2 silencing resulted in development arrest might due to the suppression of the gene expression involved in development. *EcRB1*, *USP1*, and *Broad* are involved in 20E signaling pathway [18]. The protein kinase *AKT* promotes cell proliferation and inhibits apoptosis [19]. The suppression of these genes after AK2 knockdown might be related to the larval growth arrest and development delay.

Reports state that high ADP concentration causes damage to the cells [20]. AK1 knockout in mice shows no gross abnormalities in nucleotides levels under normal condition; but under hypoxia hypoxia AK1 knockout hearts, intracellular ATP levels was decreased, Pi/ATP ratio was increased and generation of adenosine was suppressed [21]. ADP levels are accumulated during repetitive contractions of skeletal muscle in AK1 knockout mice [22]. In yeast disruption of AK gene causes accumulation of ATP in the mitochondrial intermembrane space, thus suppress adenine nucleotide translocator exported ATP in exchange for ADP [23].

Human AK2 plays an important role in providing the energy required for the proliferation of haematopoietic precursors [12]. Overexpression of AK promoted ATP production notably in yeasts [24], indicating that overexpressing AK2 supplies adequate ATP for cell proliferation. Our study shows that recombinant expressed AK2 promotes HaEpi cell growth and viability *in vitro* by MTT assay. MTT assay indicates cell growth and viability; however, it is also often used in cell proliferation detection. For example, the cell survival promoted by overexpressed AK4 under environmental stress [25] and the proliferation of MCF-7 human breast cancer cells in various treatments [26]. The *in vivo* function of *Helicoverpa* AK2 to promote cell proliferation is confirmed by the decrease in the number of circulating haemocytes after AK2 knockdown. Insect-circulating haemocytes are supplied by haemopoiesis in the haemopoietic organs or by mitotic division of a circulating stem cell [27]. AK2 knockdown decreases circulating haemocytes, suggesting that AK2 functions in promoting cell proliferation.

The recombinant expressed non-secreted proteins are able to enter the invertebrate heamocytes after being injected into the heamolymph [28], which might because the endocytosis of the invertebrate heamocytes. We found the recombinant expressed non-secreted AK2 is able to enter the cultured epidermal cells. The mechanism of the recombinant expressed protein entering the cultured cells without degradation might because that the endocytosis ability of the cells, the culture medium, or the culture temperature is different from the mammals.

## Conclusions

*H. armigera* AK2 shows high identity to other AK2 from various animals. AK2 is essential to the larval growth and development. The function of AK2 is to promote cell growth. The recombinant expressed AK2 in *E. coli* is able to promote cell growth and viability *in vitro*. AK2 is necessary for maintaining the circulating haemocytes number. AK2 is involved in regulating mRNA levels of the genes involved in insect growth and development.

## Methods

### Experimental animal

The cotton bollworm was maintained in the lab on an artificial diet described in a previous work under a daily photoperiod of 14 h at 27°C [29].

### Bioinformatics analysis and phylogenetic tree analysis of the AK2 gene

*AK2* was obtained by sequencing the constructed cDNA library. Identity analysis was performed for *AK2* through BLASTX (http://www.ncbi.nlm.nih.gov/). The AK domain of the deduced protein was achieved using ExPASy (http://au.expasy.org). The phylogenetic tree for *AK* was produced using MEGA 3.1 (Molecular Evolutionary Genetics Analysis, Version 3.1).

### Recombinant expression of AK2 and antiserum preparation

The ORF of AK2 (726 bp) was amplified from the cDNA library using the primers AK2ExpF (5^′^-tactcaggatccatggtgcagaaaggtcct-3^′^) and AK2ExpR (5^′^-tactcactcgagttagaatcctcgaagaacagc -3^′^), with the *EcoR* I and *Xho* I restriction sites inserted at the beginning and end of ORF, respectively. The PCR products of *AK2* were obtained using the following procedures: the first cycle at 94°C for 3 min; the next 35 cycles at 94°C for 30 s, 55°C for 45 s, 72°C for 30 s; and the final cycle at 72°C for 10 min. Subsequently, the products were inserted into the restriction sites of the pET30a plasmid.

The recombinant pET30a-*AK2* plasmid was transformed into *E. coli* Rosetta host cells. The target protein was then induced by isopropyl-β-D-thiogalactopyranoside (IPTG, 0.01 mM) in kanamycin/Luria–Bertani (100 μg/ml) medium for 6 h at 28°C. Afterward, the cells were collected by centrifuging at 4000 g for 5 min, resuspended in a phosphate-buffered saline (PBS, 140 mM NaCl, 2.7 mM KCl, 10 mM Na_2_HPO_4_, and 1.8 mM KH_2_PO_4_) containing 0.2% Triton X-100, and then sonicated. The soluble recombinant AK2 protein was purified from the supernatant using His-Bind resin (Ni^2+^-resin) (Novagen, Darmstadt, Germany) following manufacturer instructions. SDS-PAGE (12.5%) was performed to analyze the purified proteins. The purified protein (100 μg) was further purified via SDS-PAGE and the target band was homogenized with 1 ml complete Freund’s adjuvant (Sigma, St Louis, MO). The mix was injected into a rabbit once a week for three weeks, and post-immune serum was collected. Western blot analysis was performed to examine antiserum specificity.

### Western blot

After SDS-PAGE, the proteins were electrically transferred onto a nitrocellulose membrane. The membrane was treated using the following procedure with shaking at room temperature: blocking with 2% nonfat dry milk in TBS for 1 h followed by incubation in antiserum against Ha-AK2 (1:100 in block solution) overnight. Subsequently, the membrane was washed in TBST (0.1% Tween-20 in TBS) for 3 × 10 min, incubated in peroxidase-conjugated goat-anti-rabbit IgG (1:10000 in blocking solution) for 4 h, followed by 3 × 10 min washes with TBST and 1 × 10 min wash using TBS. The target protein was then visualized by allowing peroxidase to react with a peroxidase-staining reaction mixture [1 ml 4-chloro-1-naphtholin methanol (6 mg/ml); 9 ml TBS; 6 μl H_2_O_2_] in the dark for 5 min–30 min.

### Semiquantitative RT-PCR

Total RNAs were isolated from the tissues using Unizol reagent. Total RNA (2 μg) was used for reverse transcription of the first-strand cDNA using M-MLV Reverse Transcriptase (Sangon, Shanghai, China). The resulting cDNAs were used as templates in PCR reactions. Three independent experiments were performed, and data were analyzed using the Quantity One software (Bio Rad, Hercules, CA, USA).

### Immunocytochemistry

HaEpi cells [15] were seeded on a cover glass in a 24-well tissue culture plate and cultured at 27°C. When the cells grew to a confluence of 80%, the medium was removed from the dish, and the prewarmed (37°C) medium containing Mitotracker probes was added (50 nM) (Invitrogen, USA). The cells were then incubated at 27°C. After 30 min, the medium was replaced and cells were washed with PBS. The cells were then fixed in 4% paraformaldehyde solution for 10 min at room temperature. After 2 × 5 min with PBS, the cells were permeabilized in PBS containing 0.2% Triton X-100 for 10 min and blocked with 2% bovine serum albumin (BSA) for 30 min at 37°C. Following blocking, cells were incubated with anti-AK2 (diluted 1:100 in blocking buffer) overnight at 4°C, and then washed 3 × 5 min with PBS and incubated with a secondary antibody (goat anti-rabbit-Alexa Fluor 488) diluted to 1:1000 at 37°C for 1 h. After washing with PBS, the cells were stained with 4^′^-6-Diamidino-2-phenylindole dihydrochloride (DAPI; AnaSpec Inc., San Jose, CA; 1 μg/ml in water) for 10 min at RT. Fluorescence was detected using an Olympus BX51 fluorescence microscope (Olympus Optical Co., Tokyo, Japan).

### MTT cell proliferation assay

HaEpi cells were seeded in a 96-well tissue culture plate and cultured at 27°C until they reached the desired confluence. Indicated concentrations of recombinantly expressed AK2 (0.078, 0.313, 1.25, 5, and 20 μM) were added, as well as VP28, which served as the control. After 72 h, the cells were treated with MTT (5 mg/ml, 20 μl/well) (Sangon, Shanghai, China) for 6 hours. Formazan was dissolved in DMSO (150 μl/well), and absorbance at 490 nm was determined. The analysis was repeated five times for each concentration.

### RNAi in larvae via bacterial feeding

Full-length AK2 was cloned by PCR using primers AK2expF2 (tactcagcggccgcatggctccagcagctcctgca) and AK2expR2 (tactcactcgagttactgggccgccctttgctt) with the *EcoR* I and *Xho* I restriction sites inserted at the beginning and end of AK2. The PCR products were then inserted into the restriction sites of the RNAi vector L4440. The constructed vector was transformed into HT115 (DE3) bacteria. The target dsRNA was induced by isopropyl-β-D-thiogalactopyranoside (IPTG, 0.5 mM) in 200 ml Luria–Bertani medium containing ampicillin (100 μg/ml) and tetracycline (12.5 mg/ml) for 4 h at 37°C to OD_600_ = 0.6. The feeding bacteria was collected from 200 ml LB via centrifugation at 4000 g for 10 min, after which it was resuspended in 1 ml sterile water for *H. armigera* feeding assay. The artificial diet was cut into 10 mm × 10 mm × 2 mm pieces, and a 50 μl suspension of the bacteria cells expressing ds*AK2* was overlaid onto each diet piece. Bacteria-expressing dsGFP was used as control and was prepared by the same methods. Diet was refreshed daily. Each treatment contained 30 larvae. Three replicates were made for statistical analysis. The processes of insect development, molting, and metamorphosis were tracked daily. Each individual was weighed using an electronic balance (0.0001 g) (Sartorius, Goettingen, Germany) at given developmental stages. Total RNA of body fat from the fifth instar larvae was extracted for RT-PCR analysis. The present phenotype was photographed using a Canon (Powershot A 610) digital camera.

### Haemocyte counting

Hemolymph with haemocytes from the larvae was diluted 50-fold by PBS with glutathione (2 mg/ml). The diluted haemocytes were distributed on the blood cell count plate, and the cell number was counted under a microscope (Olympus Optical Co., Tokyo, Japan). Each group contained 3 larvae and the count on each larva was repeated 3 times. The number of haemocytes was statistically calculated.

## Competing interests

The authors declare that they have no competing interests.

## Authors’ contributions

Ru-Ping Chen performed the experiments on larvae and drafted the manuscript, Chun-Yan Liu did the work on cell line, Hong-Lian Shao maintained HaEpi cells, Wei-Wei Zheng cloned the AK2 cDNA, Jin-Xing Wang and Xiao-Fan Zhao designed and directed the study and write the manuscript. All authors read and approved the final manuscript.

## Supplementary Material

Additional 1 **Figure S1.** Bioinformatic and phylogenetic analysis of *Helicoverpa* AK2. **A.** cDNA and deduced amino acid sequence of *AK2*. The shadowed amino acids indicate the ADK domain, in which the boxed amino acids show the ADK_lid domain. **B.** Multiple alignments of AK2 amino acid sequences with those of corresponding genes obtained from other animals. **C.** Phynogenetic analysis of AK2 from different organisms by the neighbor-joining method in MEGA. The numbers above the branches indicate bootstrap values shown as percentage, whereas the scale bar displays the number of substitutions per site. The sequences (with GenBank accession numbers) used for the analysis included *D. melanogaster* (NP_523836), *Aedes aegypti* (XP_001662844), *Danio rerio* (NP_997761), *Mus musculus* (BAE40035), *H. sapiens* (BAG58139), *Anopheles gambiae* (XP_318704), *Homo sapiens* A (NP_001616), *H. sapiens* B (NP_037543), *M. musculus* A (NP_001029138), *M. musculus* B (NP_058591), *Tribolium castaneum* (XP_972103), *Rattus norvegicus* (BAA02378), *R. norvegicus* A (NP_112248), *R. norvegicus* B (NP_001029139), and *Xenopus tropicalis* (NP_001004791). Click here for file

## References

[B1] Dzeja PP Terzic A Phosphotransfer networks and cellular energetics J Exp Biol 2003 206 Pt 12 2039 2047 12756286 10.1242/jeb.00426

[B2] Zeleznikar RJ Dzeja PP Goldberg ND Adenylate kinase-catalyzed phosphoryl transfer couples ATP utilization with its generation by glycolysis in intact muscle J Biol Chem 1995 270 13 7311 7319 10.1074/jbc.270.13.7311 7706272

[B3] Glaser M Nulty W Vagelos PR Role of adenylate kinase in the regulation of macromolecular biosynthesis in a putative mutant of Escherichia coli defective in membrane phospholipid biosynthesis J Bacteriol 1975 123 1 128 136 166976 10.1128/jb.123.1.128-136.1975 PMC235699

[B4] Konrad M Molecular analysis of the essential gene for adenylate kinase from the fission yeast *Schizosaccharomyces pombe* J Biol Chem 1993 268 15 11326 11334 8496185

[B5] Panayiotou C Solaroli N Xu Y Johansson M Karlsson A The characterization of human adenylate kinases 7 and 8 demonstrates differences in kinetic parameters and structural organization among the family of adenylate kinase isoenzymes Biochem J 2011 433 3 527 534 10.1042/BJ20101443 21080915

[B6] Khoo JC Russell PJ Isoenzymes of adenylate kinase in human tissue Biochim Biophys Acta 1972 268 1 98 101 10.1016/0005-2744(72)90202-1 5018282

[B7] Gellerich FN The role of adenylate kinase in dynamic compartmentation of adenine nucleotides in the mitochondrial intermembrane space FEBS Lett 1992 297 1–2 55 58 1551437 10.1016/0014-5793(92)80326-c

[B8] Janssen E de Groof A Wijers M Fransen J Dzeja PP Terzic A Wieringa B Adenylate kinase 1 deficiency induces molecular and structural adaptations to support muscle energy metabolism J Biol Chem 2003 278 15 12937 12945 10.1074/jbc.M211465200 12562761

[B9] Zhai R Meng G Zhao Y Liu B Zhang G Zheng X A novel nuclear-localized protein with special adenylate kinase properties from Caenorhabditis elegans FEBS Lett 2006 580 16 3811 3817 10.1016/j.febslet.2006.05.074 16781712

[B10] Pannicke U Honig M Hess I Friesen C Holzmann K Rump EM Barth TF Rojewski MT Schulz A Boehm T Reticular dysgenesis (aleukocytosis) is caused by mutations in the gene encoding mitochondrial adenylate kinase 2 Nat Genet 2009 41 1 101 105 10.1038/ng.265 19043417

[B11] Fujisawa K Murakami R Horiguchi T Noma T Adenylate kinase isozyme 2 is essential for growth and development of *Drosophila melanogaster* Comp Biochem Physiol B Biochem Mol Biol 2009 153 1 29 38 10.1016/j.cbpb.2009.01.006 19416704

[B12] Lagresle-Peyrou C Six EM Picard C Rieux-Laucat F Michel V Ditadi A Demerens-de Chappedelaine C Morillon E Valensi F Simon-Stoos KL Human adenylate kinase 2 deficiency causes a profound hematopoietic defect associated with sensorineural deafness Nat Genet 2009 41 1 106 111 10.1038/ng.278 19043416 PMC2612090

[B13] Kohler C Gahm A Noma T Nakazawa A Orrenius S Zhivotovsky B Release of adenylate kinase 2 from the mitochondrial intermembrane space during apoptosis FEBS Lett 1999 447 1 10 12 10.1016/S0014-5793(99)00251-3 10218571

[B14] Lee HJ Pyo JO Oh Y Kim HJ Hong SH Jeon YJ Kim H Cho DH Woo HN Song S AK2 activates a novel apoptotic pathway through formation of a complex with FADD and caspase-10 Nat Cell Biol 2007 9 11 1303 1310 10.1038/ncb1650 17952061

[B15] Shao HL Zheng WW Liu PC Wang Q Wang JX Zhao XF Establishment of a new cell line from lepidopteran epidermis and hormonal regulation on the genes PLoS One 2008 3 9 e3127 10.1371/journal.pone.0003127 18769621 PMC2518862

[B16] Burkart A Shi X Chouinard M Corvera S Adenylate kinase 2 links mitochondrial energy metabolism to the induction of the unfolded protein response J Biol Chem 2011 286 6 4081 4089 10.1074/jbc.M110.134106 20876536 PMC3039340

[B17] Noma T Murakami R Yamashiro Y Fujisawa K Inouye S Nakazawa A cDNA cloning and chromosomal mapping of the gene encoding adenylate kinase 2 from *Drosophila melanogaster* Biochim Biophys Acta 2000 1490 1–2 109 114 10786623 10.1016/s0167-4781(99)00223-7

[B18] Riddiford LM Hiruma K Zhou XF Nelson CA Insights into the molecular basis of the hormonal control of molting and metamorphosis from Manduca sexta and *Drosophila melanogaster* Insect Biochem Molec 2003 33 12 1327 1338 10.1016/j.ibmb.2003.06.001 14599504

[B19] Zhang X Tang N Hadden TJ Rishi AK Akt, FoxO and regulation of apoptosis Biochim Biophys Acta 2011 1813 11 1978 1986 10.1016/j.bbamcr.2011.03.010 21440011

[B20] Heine P Braun N Heilbronn A Zimmermann H Functional characterization of rat ecto-ATPase and ecto-ATP diphosphohydrolase after heterologous expression in CHO cells Eur J Biochem 1999 262 1 102 107 10.1046/j.1432-1327.1999.00347.x 10231370

[B21] Pucar D Janssen E Dzeja PP Juranic N Macura S Wieringa B Terzic A Compromised energetics in the adenylate kinase AK1 gene knockout heart under metabolic stress J Biol Chem 2000 275 52 41424 41429 10.1074/jbc.M007903200 11006295

[B22] Hancock CR Brault JJ Wiseman RW Terjung RL Meyer RA 31P-NMR observation of free ADP during fatiguing, repetitive contractions of murine skeletal muscle lacking AK1 Am J Physiol Cell Physiol 2005 288 6 C1298 1304 10.1152/ajpcell.00621.2004 15689408

[B23] Bandlow W Strobel G Zoglowek C Oechsner U Magdolen V Yeast adenylate kinase is active simultaneously in mitochondria and cytoplasm and is required for non-fermentative growth Eur J Biochem 1988 178 2 451 457 10.1111/j.1432-1033.1988.tb14469.x 2850178

[B24] Sakai Y Rogi T Yonehara T Kato N Tani Y High-level ATP production by a genetically-engineered *Candida yeast* Biotechnology (N Y) 1994 12 3 291 293 10.1038/nbt0394-291 7764491

[B25] Liu R Strom AL Zhai J Gal J Bao S Gong W Zhu H Enzymatically inactive adenylate kinase 4 interacts with mitochondrial ADP/ATP translocase Int J Biochem Cell Biol 2009 41 6 1371 1380 10.1016/j.biocel.2008.12.002 19130895 PMC2676352

[B26] Duo J Ying GG Wang GW Zhang L Quercetin inhibits human breast cancer cell proliferation and induces apoptosis via Bcl-2 and Bax regulation Mol Med Report 2012 5 6 1453 1456 22447039 10.3892/mmr.2012.845

[B27] Yamashita M Iwabuchi K Bombyx mori prohemocyte division and differentiation in individual microcultures J Insect Physiol 2001 47 4–5 325 331 11166296 10.1016/s0022-1910(00)00144-x

[B28] Chen AJ Wang S Zhao XF Yu XQ Wang JX Enzyme E2 from Chinese white shrimp inhibits replication of white spot syndrome virus and ubiquitinates its RING domain proteins J Virol 2011 85 16 8069 8079 10.1128/JVI.00487-11 21680526 PMC3147975

[B29] Zhao XF Wang JX Xu XL Schmid R Wieczorek H Molecular cloning and characterization of the cathepsin B-like proteinase from the cotton boll worm, *Helicoverpa armigera* Insect Mol Biol 2002 11 6 567 575 10.1046/j.1365-2583.2002.00366.x 12421414

