# Supplementary figures and images for: Adenylate kinase 2 (AK2) promotes cell proliferation in insect development
Source: BMC Mol Biol. 2012 Sep 28;13:31. doi: 10.1186/1471-2199-13-31 (PMC3583204; doi:10.1186/1471-2199-13-31)

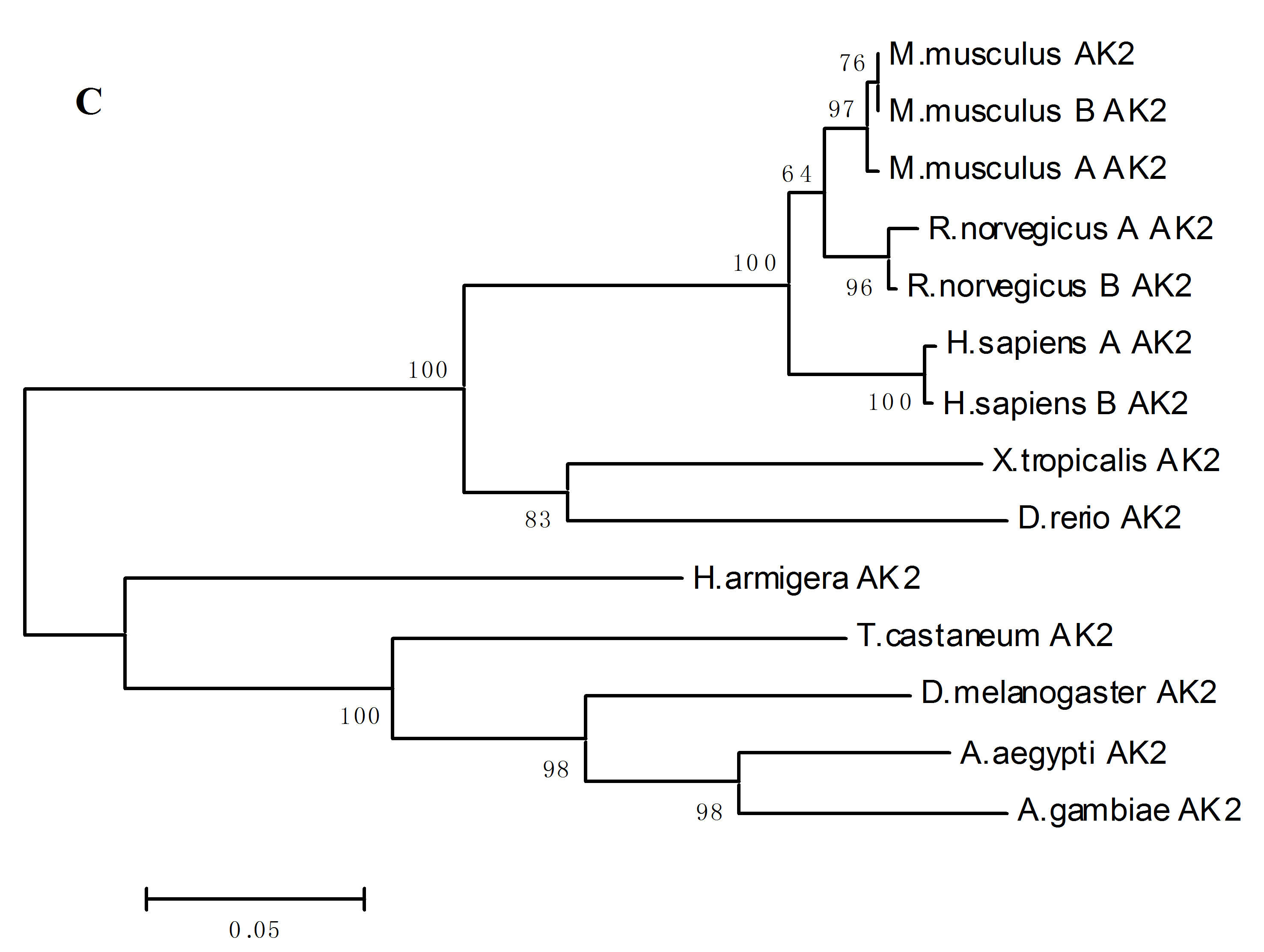

Supplement: Additional 1 — Figure S1. Bioinformatic and phylogenetic analysis of Helicoverpa AK2. A. cDNA and deduced amino acid sequence of AK2. The shadowed amino acids indicate the ADK domain, in which the boxed amino acids show the ADK_lid domain. B. Multiple alignments of AK2 amino acid sequences with those of corresponding genes obtained from other animals. C. Phynogenetic analysis of AK2 from different organisms by the neighbor-joining method in MEGA. The numbers above the branches indicate bootstrap values shown as percentage, whereas the scale bar displays the number of substitutions per site. The sequences (with GenBank accession numbers) used for the analysis included D. melanogaster (NP_523836), Aedes aegypti (XP_001662844), Danio rerio (NP_997761), Mus musculus (BAE40035), H. sapiens (BAG58139), Anopheles gambiae (XP_318704), Homo sapiens A (NP_001616), H. sapiens B (NP_037543), M. musculus A (NP_001029138), M. musculus B (NP_058591), Tribolium castaneum (XP_972103), Rattus norvegicus (BAA02378), R. norvegicus A (NP_112248), R. norvegicus B (NP_001029139), and Xenopus tropicalis (NP_001004791). [file 1471-2199-13-31-S1.tiff]
